# The cultural currency of Nicholas Cage: Does it matter for teaching medicine?

**DOI:** 10.1111/medu.15744

**Published:** 2025-08-08

**Authors:** Pip Garner, Stacy‐Paul Wilshaw, Matthew E. Hardy

**Affiliations:** ^1^ School of Pharmacy and Medical Sciences, Faculty of Life Sciences University of Bradford Bradford UK

## Abstract

Medical educators are often encouraged to develop innovative teaching practices. This can include using media such as movies and film clips to illustrate good or bad science and/or medical practice. As material gets older, educators are encouraged to find newer or more culturally aligned content, in order to stay relevant. This playful contribution to Medical Education Unleashed questions the necessity of updating media used in teaching to relate to students. We used a film clip from Michael Bay's 1996 movie, ‘The Rock’, and instructed students to fact check what Nicholas Cage tells us about Vx nerve agent—they could associate this with synapses such as the neuromuscular junction. On surveying the students, we found the majority of them perceived that the clip helped them with understanding and remembering information, as well as focusing during the workshop. This was despite none of them having been born at the time the film was released, and some being unfamiliar with the film, or its stars: Sean Connery and Nicholas Cage. Using this small, statistically nonviable study, we can conclude that film media can still be used for educational benefit, regardless of how well it aligns with the age and culture of students.

## Abstract 2

Nicholas Cage never gets old! Even 30 years later, the film ‘The Rock’ offers benefit for teaching students about the Neuromuscular Junction! #MedEducUnleashed

## 1 INTRODUCTION

Cinemeducation is a concept that combines the use of movies/clips with the teaching of medicine.^1^ There are crucial learning aspects that suggest value to using film in medical education. For example, learning to observe and listen to patients may be favourably compared to using the sounds and visual on a cinema screen to carefully parse the messages being conveyed, which are often subtle and easy to miss.^2^ Often these educational approaches make use of person‐to‐person dynamics within a film; if an on‐screen healthcare provider fails to identify cues regarding concerns a patient may have, this may be used as a learning experience for students.

In determining how to enable such benefits, it has been suggested that older films may present with outdated medical interactions and technology.^3^ Given changes in movie‐making economics, movies of the action and adventure genres have increased to account for more than half the box‐office revenue in the United States.^4^ This raises similar questions about whether Hollywood action movies should be used to teach medical science? When Sylvester Stallone cauterised a bullet wound with gunpowder,^5^ it is unlikely that many medical educators paused to consider whether this was a teachable moment (Note: Authors 1 and 2 would like to add: please do not teach students how to cauterise wounds with gunpowder; this is neither safe nor approved medical practice, no matter how cool author 3 thinks it is). Those that do consider such questions should also consider whether using scenes from Rambo 3 is the best approach for all students; some may not like action movies or anything with special effects that predates computer generated imagery (CGI). Can the student who prefers Bollywood musicals, K dramas or even romcoms really learn anything from Sylvester Stallone? Further, there is always the risk that when presenting such innovative educational practices at conferences, some rival academic may disparage this work as being culturally exclusive.

To address these issues, this study aimed to elucidate whether educators can teach biology and medicine effectively using the ‘science’ in Hollywood action movies, while simultaneously disregarding the age and cultural relevance of the material in relation to the students.

.

## 2 METHODS

We spent a considerable amount of time watching movies to identify film clips with a modicum of useful medical information. For this reason, we discounted the use of any Sylvester Stallone movies and identified the classic 1996 action movie: The Rock, in which Sean Connery and Nicholas Cage starred.^6^ The Rock contains scientific content relating to Vx nerve agent poisoning contained in a short extract. Nicholas Cage (in the character of toxicology expert Stanley Goodspeed) explains the effects of Vx nerve agent to a horrified Sean Connery (ex‐SAS soldier John Mason). Using this extract from ‘The Rock’ also appealed to author 3 because having the chance to teach with anything that has Nicholas Cage in it would make him seem ‘cool and wacky’.

The relevant clip occurs at 1 hour 27 minutes into the film and lasts for approximately 2 minutes. During the ‘disarming’ of a warhead containing Vx nerve agent, Stanley Goodspeed notes how unstable the ‘elegant string of pearls configuration’ is—a preposterously fragile string of fragile glass balls containing Vx gas that no scientist, no matter how evil their aims, would ever use to store a lethal compound. He then lists the effects of the toxin (see Table 1).

**TABLE 1:** Fact checking the effects of Vx nerve agent as listed by Nicholas Cage (Stanley Goodspeed) in the 1996 movie: ‘The Rock’.

Nicholas Cage states:	Fact‐check
Cholinesterase inhibitor	Correct
Stops brain from sending signals down the spinal chord	Eventually at death (quite the opposite initially)
Twinge at small of back	? Quite likely as muscle spasms begin
Muscles ‘freeze’	Correct—uninhibited stimulation at the neuromuscular junction will cause tetanus
Can't breathe	Correct
Spasm so hard you ‘break your own back’	Spasms certainly—we have not found a case where anyone has broken their back in this manner
Spit guts out	Vomiting certainly, but the guts will remain where they are
Skins melts off	That's just ridiculous

*Note*: Effects have been checked at the Centers for Disease Control and Prevention.^7^

We used this list as an educational fact checking exercise for students to undertake during a workshop that followed lectures on physiology and transport mechanisms, as well as including details regarding synapses such as the muscarinic and nicotinic receptors in the autonomic nervous system, and the neuromuscular junction. Using the knowledge that Vx nerve agent inhibits acetylcholinesterase, thus causing acetylcholine to accumulate and cause ongoing stimulation at the relevant synapse, students were expected to ascertain whether Stanley Goodspeed was listing facts or Hollywood fiction. Following this, we corrected the students in the hope they would learn something as well as providing them with a more accurate list of the effects.^7^

Having used the clip as a teaching aid in a Foundation Clinical Sciences programme for several years, we finally decided we should do some ‘actual research’ by surveying our students. Questionnaires were handed out to students during the workshop. Students were informed that their responses were anonymous and (despite us knowing full well the impact on response rate of telling them this) that their participation was optional (Data S1). Surveys and collection methods were reviewed and approved by the Chair of the Biomedical, Natural, Physical and Health Sciences Research Ethics Panel at the University of Bradford.

## 3 RESULTS

Of the 63 students who attended the workshop, 18 could be bothered to complete the survey. Of these, one set of responses had to be discarded because the student could not fill in the text boxes appropriately. The demographics recorded from the remaining 17 surveys can be seen in Table 2. Respondents primarily identified themselves as British or British Pakistani (14 students), female (12 students) and Asian or Black (14 and 3, respectively). This aligns with the dissimilarity in enrolment demographic at The University of Bradford (compared to the rest of the United Kingdom) that has previously been observed^4^ and may suggest that the students who answered the survey were less likely to find cultural familiarity with a predominantly White, western movie.

**TABLE 2:** Demographic of students who responded to the survey.

Characteristic	Student ratio
Nationality (British:British‐Pakistani:Pakistani:German:Spanish)	11:2:2:1:1
Gender (female:male)	12:5
Ethnicity (Asian:Black)	14:3
Age (15–20 years:21–25 years)	15:2
Has a preference for action movies (yes:no)	13:4

Survey questions assessed student perceptions of whether the film clip helped them to stay focused during the workshop, whether they would remember and understand content and whether correcting errors and identifying correct facts would aid in their understanding. These were also considered in relation to students' perceived film and cultural preferences. No students thought that the use of a Hollywood movie clip was inappropriate for teaching with.

**FIGURE 1:**
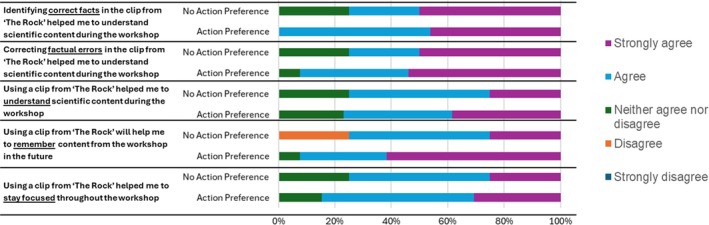
The impact of film preference (action films) on the effectiveness of using a film clip from ‘The Rock’ in science education. Survey answers (n = 17) are used to determine whether familiarity influenced: students perception of staying focused; remembering and understanding content; and if correcting or identifying factual errors helped to understand content. Four out of seventeen students did not display a preference for action films. Only one student, who did not have a preference for action films, disagreed the clip helped in remembering content, whereas the majority of students perceived a positive impact in all areas. [Color figure can be viewed at wileyonlinelibrary.com]

Figure 1 divides the students into groups who showed a preference for action movies (13 students) versus those who did not (4 students). These data have cleverly been displayed as percentages so it appears as though the sample sizes are similar. The majority of respondents did not appear to be negatively influenced by genre preference when ascertaining their learning.

**FIGURE 2:**
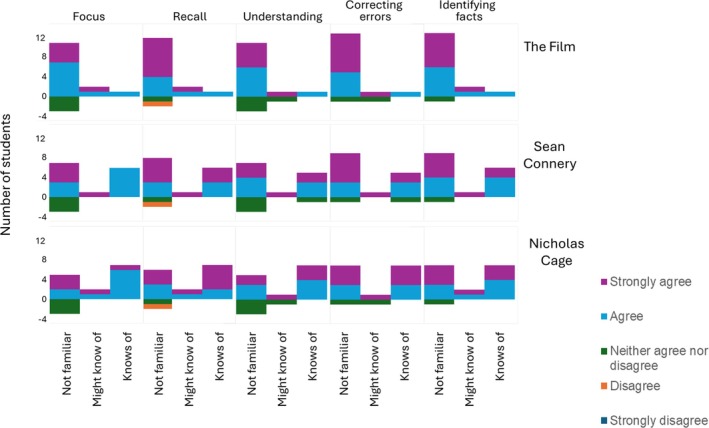
The impact of familiarity with the film ‘The Rock’ and its starring actors, Sean Connery and Nicholas Cage, on the effectiveness of using a film clip in science education. Survey answers (n = 17) are used to determine whether familiarity (x‐axis) influenced: students perception of staying focused; remembering and understanding content; and if correcting or identifying factual errors helped to understand content. Students who neither agreed nor disagreed, disagreed or disagreed strongly that familiarity helped in these characteristics are represented as negative values on the y‐axis. Regardless of familiarity with the film or leading actors, the majority of students perception was positive regarding each of the elements surveyed. Only one student, who was not familiar with the film or actors, disagreed the clip helped in remembering content. [Color figure can be viewed at wileyonlinelibrary.com]

**FIGURE 3:**
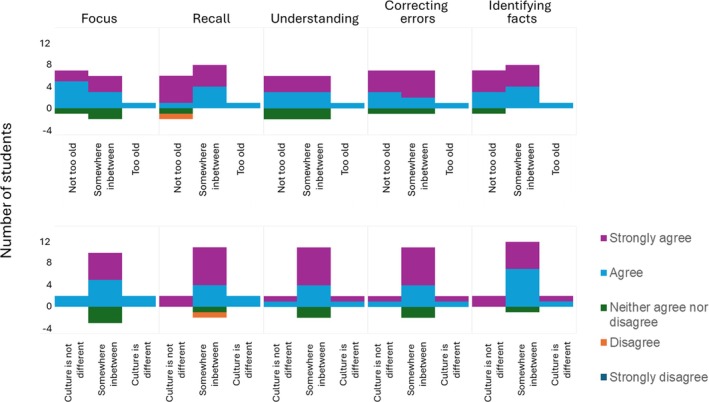
The impact of age and culture when using ‘The Rock’ in science education. None of the students were born when ‘The Rock’ was released in 1996. Survey answers (n = 17) are used to determine whether familiarity (x‐axis) influenced: students perception of staying focused; remembering and understanding content; and if correcting or identifying factual errors helped to understand content. Students who neither agreed nor disagreed, disagreed or disagreed strongly that age or culture helped in these characteristics are represented as negative values on the y‐axis. Regardless of familiarity with the film or leading actors, the majority of students perception was positive regarding each of the elements surveyed. Only one student, who neither agreed or disagreed The Rock is culturally different, but did not feel it was too old, disagreed the clip helped in remembering content. [Color figure can be viewed at wileyonlinelibrary.com]

Only one student agreed they were familiar with the film, whereas nearly half were familiar with Sean Connery (six students) and seven students were familiar with Nicholas Cage. Regardless of familiarity with the film or either actor, the majority of students still agreed that the impact of using the clip was positive in all the aspects of learning addressed in the survey questions (Figure 2). Students' impressions were similarly unrelated to whether ‘The Rock’ was of cultural relevance to them (Figure 3).

## 4 DISCUSSION

Regardless of which aspect of learning we evaluated, the majority of students agreed that the workshop helped them to focus, remember and understand content, although there was a minority who were undecided in some areas. This was generally true whether they were familiar with the film, the actors, or found them culturally or chronologically relevant. There was only one negative report which came from a single respondent who disagreed that using the movie clip would help them to remember content. This student also neither agreed nor disagreed with any other aspects of learning, remembering and understanding the workshop content. Of note, this student was not familiar with ‘The Rock’, Nicholas Cage or Sean Connery, but strongly disagreed that the film was too old to be of relevance to them.

The authors were dismayed to find that all of the students who responded to the survey were born after the film was released, and now they feel old. Author 3 was further astounded to discover that some students do not like action movies. Based on the evidence presented in this statistically nonviable dataset, however, we can conclude that using a clip from an action movie can still be perceived as beneficial by students for their understanding, retention of knowledge and focus during a workshop. This is despite delivery to students who may not automatically align with the movie as a consequence of age, culture or familiarity with the film or its stars. Therefore, the next time a rival suggests that your film clip is not suitable for your cohort, this study may be used as evidence to the contrary.

Given the small sample, we encourage further study to ascertain whether being undecided on familiarity with Nicholas cage or his movies, is detrimental to learning as well as whether a Hollywood action star can teach medicine, given that Author 3 is adamant that Nicholas Cage can do anything.

## AUTHOR CONTRIBUTIONS

**Pip Garner:** Conceptualization; investigation; methodology. **Stacy‐Paul Wilshaw:** Conceptualization; investigation; methodology. **Matthew E. Hardy:** Methodology; writing – original draft; writing – review and editing; conceptualization; investigation; formal analysis; project administration; data curation.

## CONFLICT OF INTEREST STATEMENT

The authors are not aware of any conflict of interest.

## Supporting information


**Data S1.** Supporting information

## Data Availability

The data that support the findings of this study are available from the corresponding author upon reasonable request.

## References

[1] Kadeangadi DM , Mudigunda SS . Cinemeducation: using films to teach medical students. Medknow. 2019;73‐74.

[2] Darbyshire D , Baker P . A systematic review and thematic analysis of cinema in medical education. Med Humanit. 2012;38(1):28‐33. doi:10.1136/medhum-2011-010026 22282424

[3] Hanna DR . Using motion picture films to teach nursing theory in graduate nursing education. Nurs Educ Perspect. 2019;40(4):259‐260. doi:10.1097/01.NEP.0000000000000355 29994891

[4] Leung TC , Qi S . Globalization and the rise of action movies in Hollywood. J Cult Econ. 2022;47(1):31‐39. doi:10.1007/s10824-021-09438-z

[5] MacDonald P. Rambo III. 1988

[6] Bay, M. The Rock. 1996.

[7] Centers for Disease Control and Prevention . Vx 2024. https://www.cdc.gov/chemical-emergencies/chemical-fact-sheets/vx.html

