# Supplementary material for: The cultural currency of Nicholas Cage: Does it matter for teaching medicine?
Source: Med Educ. 2025 Aug 8;60(1):34–8. doi: 10.1111/medu.15744 (PMC12717488; doi:10.1111/medu.15744)
Supplement: Supplementary file 1 — Data S1. Supporting information [file MEDU-60-34-s001.pdf]

Questionnaire on the use of media in teaching, University of Bradford, 02-2024.

Completion of this survey, in whole or in part is <u>optional</u>. All responses shall be anonymous. Data collected will be kept in a locked cabinet within an office, or password protected if stored digitally, for the duration of the project and to meet any requirements by journals should study outcomes be published.

This study has been reviewed by the Chair of the Biomedical, Natural, Physical and Health Sciences Research Ethics Panel at the University of Bradford.

A. About you

1. What is your nationality?: ______________________________

2. What is your gender?

Female	Male	Non-binary	Transgender	Prefer to self-describe	Prefer not to say
☐	☐	☐	☐	☐	☐

If you prefer to self-describe: ______________________________

3. What is your ethnic group?

White	Mixed/Multiple Ethnic Groups	Asian	Black	Other	Prefer not to say
☐	☐	☐	☐	☐	☐

If other please describe: ______________________________

4. In which age category are you?

15-20 years	21-25 years	26-30 years	31-40 years	Older than 40	Prefer not to say
☐	☐	☐	☐	☐	☐

5. My preferred genres of television/films are (tick all that apply):

Action	☐	Romance	☐
Western	☐	Romantic Comedy	☐
Crime	☐	Comedy	☐
Thriller	☐	Biography/Factual	☐
Horror	☐	Foreign Language	☐
Fantasy	☐	Historical	☐
Science Fiction	☐	War	☐
Animation	☐	Other	☐

If other please state: ______________________________

B. Your perceptions regarding the workshop and media.

PLEASE TICK THE MOST APPROPRIATE BOX FOR EACH QUESTION.

		Strongly disagree	Disagree	Neither Agree nor Disagree	Agree	Strongly agree
6	Using a clip from 'The Rock' helped me to stay focused throughout the workshop	☐	☐	☐	☐	☐
7	Using a clip from 'The Rock' will help me to remember content from the workshop in the future	☐	☐	☐	☐	☐
8	Using a clip from 'The Rock' helped me to understand scientific content during the workshop	☐	☐	☐	☐	☐
9	Correcting factual errors in the clip from 'The Rock' helped me to understand scientific content during the workshop	☐	☐	☐	☐	☐
10	Identifying correct facts in the clip from 'The Rock' helped me to understand scientific content during the workshop	☐	☐	☐	☐	☐
11	Using clips from Hollywood movies such as 'The Rock' is appropriate for teaching	☐	☐	☐	☐	☐
12	*Before the workshop, I was familiar with the film 'The Rock'	☐	☐	☐	☐	☐
13	**Before the workshop, I was familiar with the actor Sean Connery	☐	☐	☐	☐	☐
14	**Before the workshop, I was familiar with the actor Nicholas Cage	☐	☐	☐	☐	☐
15	The film 'The Rock' is too old to be relevant to me	☐	☐	☐	☐	☐
16	The film 'The Rock' is set in a culture different to my own	☐	☐	☐	☐	☐

**where 'Strongly disagree' = I had not heard of the film; and 'Strongly agree' = I have seen the film on multiple occasions and am very familiar with the plot and dialogue*

***where 'Strongly disagree' = I had not heard of him; and 'Strongly agree' = I have seen him in multiple roles and will watch material because he is in it*

Any further comments/feedback:

____

____

____
